# Residual effect of sequential 4-channel neuromuscular electrical stimulation evaluated by high-resolution manometry

**DOI:** 10.1186/s12938-024-01269-1

**Published:** 2024-07-25

**Authors:** Jiwoon Lim, Sung Eun Hyun, Hayoung Kim, Ju Seok Ryu

**Affiliations:** 1 Department of Rehabilitation Medicine, Korea University Ansan Hospital, Ansan-Si, South Korea; 2 Department of Rehabilitation Medicine, Seoul National University Hospital, Seoul, South Korea; 3 Department of Rehabilitation Medicine, Seoul National University Bundang Hospital, 82 Gumi-Ro 173 Beon-Gil, Bundang-Gu, Seongnam-Si, Gyeonggi-Do 463-707 South Korea; 4 Department of Rehabilitation Medicine, Seoul National University College of Medicine, Seoul, South Korea

**Keywords:** Dysphagia, Deglutition, Electrical stimulation, High-resolution manometry

## Abstract

**Background:**

High-resolution manometry (HRM) can quantify swallowing pathophysiology to evaluate the status of the pharynx. Sequential 4-channel neuromuscular electrical stimulation (NMES) was recently developed based on the normal contractile sequences of swallowing-related muscles. This study aimed to examine the effects of sequential 4-channel NMES for compensatory application during swallowing and to observe the residual effects after the application of NMES using HRM.

**Results:**

Sequential 4-channel NMES significantly improved the HRM parameters, with respect to the maximal pressure and area of the velopharynx (VP), maximal pressure and area of the mesopharynx (MP), and upper esophageal sphincter (UES) activation and nadir duration. Furthermore, the improvement in the pressure and area variables of the VP and MP showed a tendency to maintain even when measured after NMES, but there are no significant differences.

**Conclusions:**

The present study suggests that the sequential 4-channel NMES application of the suprahyoid and infrahyoid muscles during swallowing improves the pressure, area, and time variables of the oropharynx, as measured by HRM, and it is likely that the effects may persist even after stimulation.

*Trial Registration* Clinicaltrials.gov, registration number: NCT02718963 (initial release: 03/20/2016, actual study completion date: 06/24/2016, last release: 10/20/2020).

## Introduction

Dysphagia is a difficulty in swallowing that can occur in any of the oral, pharyngeal, or esophageal phases of swallowing [1]. It is a common symptom in many disorders and old age, and leads to various clinical complications, such as malnutrition, dehydration, and risk factors, such as aspiration pneumonia, asphyxiation, and premature death [2]. There are diverse methods for dysphagia treatment, such as oropharyngeal exercise, compensatory maneuvers, neuromuscular electrical stimulation (NMES), and diet control [3]. Especially, 2-channel NMES is one of the most common methods, and has been reported to improve swallowing ability [4–7]. In previous systemic reviews in patients with poststroke dysphagia, traditional dysphagia treatment with NMES was seen to be more effective than that without NMES or usual care without swallowing treatment, but the effectiveness of treatment with NMES alone is unclear [8–10]. Moreover, the precise mechanism of conventional 2-channel NMES treatment is unknown, and controversy remains over its efficacy and methods of stimulation [11].

Most clinical studies regarding NMES have focused on rehabilitative mechanism (cumulative effects) by facilitating muscle strengthening or swallowing reflex. [9] Hypothetically, NMES could be used not only for the rehabilitative mechanism (cumulative effects), but also for compensating for any abnormal muscle activation during NMES (during NMES effects) [9, 12, 13]. In our previous study, the suprahyoid muscles were activated approximately 300 ms before the infrahyoid muscles. The sequential contraction of these swallowing-related muscles induces a circular movement of the hyoid bone during normal swallowing, which initially moves forward-upwardly and then backward-downwardly [14, 15]. However, 2-channel NMES concurrently stimulates the suprahyoid and infrahyoid muscles, and is not based on normal physiological contractile sequences. The co-stimulation of these muscles via the 2-channel NMES may result in hyolaryngeal descent and cancellation of the positive effect [16, 17]. Unlike the conventional NMEs, the stimulation these muscle via the sequential 4-channel NMES may lead to a better modification of the abnormal hyoid and laryngeal motion. Therefore, using 4-channel NMES, we devised more effective and functional stimulation methods, which stimulate these muscles in a similar manner to the normal contractile sequence during swallowing. In our previous randomized clinical trial, the compensatory and cumulative application of sequential 4-channel NMES resulted in clinical and kinematic improvements in patients with dysphagia [13, 18]. However, the residual effect of sequential 4-channel NMES, which is the retention effect after the immediate withdrawal of the stimulation, remains unclear.

Swallowing is a pressure-driven process; accordingly, detailed measurement and analysis of the changes in pressure is essential to understand the swallowing process and to find out the pathophysiology of dysphagia [19, 20]. Furthermore, it was demonstrated that the pharyngeal pressure may be altered by NMES [21, 22]. The videofluroscopic swallowing study (VFSS) is the gold standard for the diagnosis of dysphagia. However, VFSS can only identify the movement of anatomic structures and bolus, without the quantitative analysis of pharyngeal pressure changes during swallowing. High-resolution manometry (HRM), an evaluation tool that is emerging in clinical dysphagia practice, objectively identifies abnormalities in pharyngeal function by quantifying pressure changes across the pharynx. It also improves sensitivity, reliability, and accuracy [23–25]. Our previous study identified significant HRM parameters that are highly specific for individual abnormalities in VFSS. [26].

Based on previous findings, we hypothesized that the compensatory application of sequential 4-channel NMES on the suprahyoid and infrahyoid muscles may lead to changes in pharyngeal pressure and UES opening, and have residual effects after the stimulation. This study aimed to use HRM to examine the effects of sequential 4-channel NMES for compensatory application during swallowing and to investigate the residual effects persisting even after the application of NMES.

## Results

Table 1 shows the changes of HRM parameters between pre- and during NMES in each group. When swallowing during NMES in the healthy group, the VP maximal pressure, VP area and MP area were significantly greater than pre-NMES (*P* < 0.05). Moreover, when comparing between pre- and during NMES in the patient group, a significant increase in VP maximal pressure was observed (*P* < 0.05).

**Table 1:** Comparisons of HRM parameters between pre-NMES and during NMES

HRM parameters	Patient			Healthy		
	Pre-NMES	During NMES	*P* value	Pre-NMES	During NMES	*P* value
VP maximal pressure	**120.03 (79.84)**	**150.98 (87.68)**	0.015*	**130.96 (60.73)**	**185.38 (53.77)**	0.008*
VP area	25.75 (25.83)	27.99 (16.42)	0.89	**29.43 (15.16)**	**56.39 (28.78)**	0.006*
Onset time of VP first peak	0.96 (0.10)	1.19 (0.23)	0.23	0.74 (0.11)	0.80 (0.15)	0.39
MP maximal pressure	82.84 (61.44)	103.23 (73.48)	0.31	110.20 (59.60)	144.91 (30.03)	0.11
MP area	34.00 (30.86)	33.25 (31.90)	0.88	**28.74 (23.16)**	**57.25 (21.13)**	0.03*
IPC maximal pressure	215.25 (140.17)	197.78 (136.78)	0.32	304.68 (95.41)	363.96 (123.29)	0.09
Pre-UES maximal pressure	98.24 (67.13)	101.81 (52.13)	0.75	215.09 (62.74)	187.89 (69.02)	0.39
Pre-UES area	19.25 (17.29)	29.25 (36.43)	0.46	38.50 (13.77)	33.96 (15.48)	0.58
UES maximal pressure	149.89 (103.48)	178.08 (85.18)	0.12	272.00 (112.45)	235.65 (85.04)	0.35
UES minimal pressure	− 8.70 (1.62)	− 13.30 (2.78)	0.11	− 13.09 (9.02)	− 13.75 (6.06)	0.87
UES activation time	0.85 (0.06)	1.03 (0.27)	0.23	0.60 (0.09)	0.65 (0.15)	0.30
UES nadir duration	0.38 (0.03)	0.53 (0.16)	0.11	0.30 (0.08)	0.34 (0.10)	0.12
Time interval, VP onset—pre-UES	0.13 (0.05)	0.16 (0.13)	0.49	0.1 (0.06)	0.12 (0.07)	0.39

The bold values indicate statistically significant results

Values are expressed as mean (SD)

*P < 0.05

**Fig. 1:**
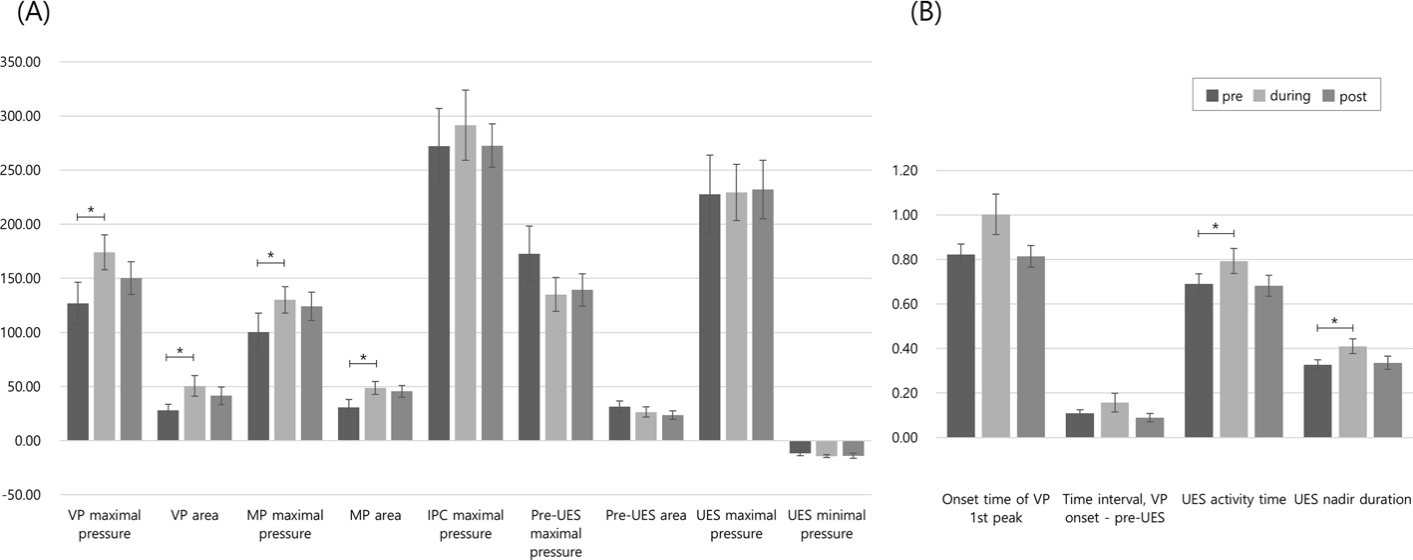
Comparison of high-resolution manometry (HRM) parameters among pre-, during, and post-NMES. **A** Among the visuospatial (pressure and area) parameters of HRM, VP maximal pressure, VP area, MP maximal pressure, and MP area were significantly improved in swallowing, concurrent with NMES. **B** Among the time variables of HRM, the UES activation and nadir duration was significantly improved in swallowing with NMES. Abbreviations: VP: velopharynx; MP: mesopharynx; IPC: Inferior pharyngeal constrictor; UES: upper esophageal sphincter..*: *P* < 0.05

Changes in the evaluated variables pre-, during, and post-NMES in all participants are presented in Table 2 and Fig. 1. Based on a linear mixed model, there were no interactions between group and time factors (*P* > 0.05). Therefore, the model including the no interaction was used as the final model. When swallowing was concurrent with NMES, the VP maximal pressure, VP area, MP maximal pressure, and MP area were significantly greater than pre-NMES (*P* < 0.05). Among the timing variables, the UES activation and nadir duration were significantly improved when swallowing was concurrent with NMES.

**Table 2:** Comparisons of HRM parameters among pre-NMES, during NMES, and post-NMES in whole participants

HRM parameters	Pre-NMES	During NMES	Post-NMES	P value	
				Pre vs. During	Pre vs. Post
VP maximal pressure	**126.98 (64.46)**	174.07 (67.95)*	150.38 (56.30)	< 0.001*	0.08
VP area	**28.09 (18.48)**	50.62 (39.77)*	41.50 (30.53)	0.01*	0.40
Onset time of VP first peak	0.82 (0.15)	1.00 (0.39)	0.81 (0.18)	0.10	0.90
MP maximal pressure	**100.25 (58.77)**	130.19 (51.77)*	124.10 (48.72)	0.02*	0.14
MP area	**30.65 (24.79)**	48.88 (25.01)*	45.57 (20.72)	0.01*	0.07
IPC maximal pressure	272.16 (115.72)	291.51 (137.82)	272.65 (75.38)	0.30	0.60
Pre-UES maximal pressure	172.60 (84.79)	135.07 (66.06)	139.27 (56.13)	0.12	0.06
Pre-UES area	31.50 (17.26)	26.46 (20.35)	23.61 (14.41)	0.70	0.30
UES maximal pressure	227.60 (120.81)	229.42 (110.04)	232.13 (101.53)	0.80	0.40
UES minimal pressure	− 11.49 (7.38)	− 14.22 (6.17)	− 13.95 (8.93)	0.30	0.50
UES activation time	**0.69 (0.15)**	0.79 (0.24)*	0.68 (0.18)	0.03*	0.50
UES nadir duration	**0.33 (0.08)**	0.41 (0.14)*	0.34 (0.11)	0.03*	0.20
Time interval, VP onset—pre-UES	0.11 (0.05)	0.16 (0.18)	0.09 (0.07)	0.30	0.60

The bold values indicate statistically significant results

Values are expressed as mean (SD)

*P < 0.05

When comparing HRM parameters before and immediately after NMES to confirm the residual effect, the VP maximal pressure and area increased from 126.98 to 150.38 (regression coefficient 22, 95% CI − 2.4 to 46.4) and 28.09 to 41.50 (regression coefficient 8.12, 95% CI − 9.6 to 25.8), respectively. Likewise, the MP maximal pressure and area increased from 100.25 to 124.10 (regression coefficient 21.12, 95% CI − 7.3 to 49.6) and 30.65 to 45.57 (regression coefficient 14.2, 95% CI − 1.3 to 29.7), respectively. However, the differences were not statistically significant.

## Discussion

This study showed that the sequential 4-channel NMES activating the suprahyoid and infrahyoid muscles at appropriate intervals during swallowing might have a residual effect. The pressure and area of VP and MP measured by HRM, showed a significant increase during swallowing with sequential 4-channel NMES; this result showed a tendency to persist even after electrical stimulation. NMES, a substitute for voluntary contraction, may be used to restore and increase muscle strength as well as enable muscle contraction [27, 28]. Previous studies have shown the effectiveness of NMES in increasing the cross-sectional area and isometric strength of type II myofibers [29, 30]. Therefore, it is presumed that the mechanism of improved swallowing with NMES is to improve swallowing-related muscle strength. Moreover, after the application of this sequential 4-ch NMES, the increased strength of the muscle might still influence even when swallowing without electrical stimulation.

HRM parameter improvements by 4-channel NMES were observed in the VP, MP, and UES regions, which are important for safe swallowing [31]. First, VP contraction prevents nasal regurgitation and is involved in the initial step of sequential peristaltic movement of the pharynx by creating a tight seal and squeezing pressure between the velum and the pharyngeal wall during swallowing [32, 33]. VP insufficiency can lead to pharyngeal pressure reduction, resulting in a lack of clearance and risk of aspiration during or after swallowing. [31, 32] Our previous study showed that maximal VP pressure was significantly correlated with VFSS parameters relevant to penetration and aspiration [24]. These results are consistent with a previous study that identified decreased VP maximal pressure as an important predictor of aspiration pneumonia in patients with oropharyngeal dysphagia [34]. Second, the MP is where a bolus is directed to the airway [35]. In addition, the strength of tongue base, located in the MP, is important for forming and manipulating the bolus in the oral phase, as well as its propulsion from the oral to the pharyngeal phases; its alteration may result in a risk of aspiration before or after swallowing [36]. Producing tongue pressure by pushing the tongue against the palate involves lifting the tongue muscles via extrinsic and intrinsic tongue muscles, and elevating the floor of the mouth via suprahyoid muscle contraction [37]. During initial squeezing, tongue pressure, hyoid movement, and suprahyoid muscle activity appeared simultaneously. When the hyoid was in an elevated position, the amplitude of suprahyoid muscle activity and tongue pressure peaked [38]. Our previous study showed that the maximal pressure and area of the TB showed a significant trend for impaired laryngeal elevation and residue in the pyriformis sinus. Third, the UES relaxation must be sufficient to allow the complete transfer of bolus from the pharyngeal cavity to the proximal esophagus. Insufficient contraction of the swallowing-related muscles leads to decreased UES opening, which reduces the passage of bolus passing into the esophagus, resulting in residue in the pyriformis sinus, supraglottic penetration, and ultimately aspiration [26, 37, 39, 40].

The stimulation algorithm of sequential 4-channel NMES is based on normal contractile sequences. In the electromyography analysis, the activations of the suprahyoid muscles developed approximately 300 ms earlier than that of the infrahyoid muscles. After 1400 ms of suprahyoid muscle contraction, all of these muscles stop contracting simultaneously [14, 15]. These sequential contractions of the suprahyoid and infrahyoid muscles cause a circular movement of the hyoid bone during normal swallowing. The thyrohyoid muscle assists in laryngeal elevation, and other infrahyoid muscles assist the opening of the UES by producing prolonged anterior motion of the hyoid bone [18, 41]. Thus, the contractions of the infrahyoid muscles, which have proper interval time with those of the suprahyoid muscles, may be important for dysphagia treatment. The results of our previous studies demonstrated the kinematic and clinical improvements of the sequential 4-channel NMES. The current results are consistent with previous findings. Sequential 4-channel NMES, based on normal contractile sequences, may improve UES relaxation. It may also improve the VP and MP strength through sustained stimulation of these muscle.

This is the first study to evaluate the residual effects of sequential 4-channel NMES using HRM. Although several studies using conventional manometry were conducted to evaluate dysphagia, this had limitations to coverage along the entire pharynx [42–45]. Because conventional manometry uses hydrostatic pressure, only limited sensors and positions (supine) were allowed; therefore, it was difficult to detect pharyngeal dysphagia [46]. We previously reported the feasibility of HRM for evaluating pharyngeal dysphagia by obtaining data on swallowing along the VP and UES. We demonstrated that this HRM was more sensitive than the kinematic analysis used in VFSS, and by measuring the precise anatomical structure, it could provide a quantitative analysis of the pressure events and timing data for pharyngeal swallowing [24, 47].

Our study had several limitations. First, it was a case–control, pilot study with a small sample size. The repeated application of NMES induces changes in muscle fiber composition, enzyme activity, and gene expression. The chronic application of NMES resulted in conversion to more slowly contracting and fatigue-resistant muscle fiber types, and improved fatigue and functional exercise capacity by increasing oxidative enzyme activity and gene expression changes [48, 30]. Further studies on these mechanisms are needed to investigate the exact compensatory and cumulative effects of 4-channel NMES. Second, the HRM measurement could be limited by high variability; therefore, two swallows and such small differences may be a confounding factor in our results. Third, the sequential four-channel NMES was not directly compared with the two-channel conventional NMES. In future studies, it is necessary to evaluate the cumulative and residual effects of sequential four-channel NMES and directly compare it with the previous, conventional two-channel NMES. Fourth, we enrolled both healthy individuals and patients with dysphagia. In addition, we could not statistically analyze the residual effect in each group because of missing values. Although this was an exploratory observational study to verify the effects of 4-channel NMES, it may be controversial. In the future, stricter inclusion criteria may be required. Fifth, there were diverse etiologies among the patients with dysphagia in our study. These differences may have affected the results. Therefore, further studies with controlled disease etiologies are necessary to determine the effectiveness of 4-channel NMES.

## Conclusions

This study suggested that the sequential 4-channel NMES application of the suprahyoid and infrahyoid muscles during swallowing improve the pressure and area variables of the oropharynx, as evaluated using HRM, and the effect may persist even after the intervention. Further prospective trials will be required to ascertain these findings.

## Methods

### Study design

To explore the residual effects as well as compensatory effects of sequential four-channel NMES, we conducted an additional analysis of data from the previous trial that evaluated the effects of four-channel NMES on swallowing kinematics and pressures [49]. This prospective clinical study was conducted between August 2015 and June 2016. The study protocol was approved by the institutional review boards of our hospital (IRB Nos.: B-1507/306-002), and all methods were performed in accordance with approved guidelines and regulations. This study was registered at clinicaltrial.gov (registration number; NCT02718963, initial release: 03/20/2016, actual study completion date: 06/24/2016, last release: 10/20/2020). All participants provided written informed consent before participation.

### Participants

One healthy individual and one patient with dysphagia, who did not perform dry swallowing before HRM evaluation, were excluded. A total of eighteen participants (nine healthy individuals and nine patients with dysphagia) over 18 years of age were enrolled in the study. The healthy individuals had no history of disease that could cause dysphagia and no symptoms of dysphagia. The inclusion criteria for patients with dysphagia were as follows: (1) at least one symptom of dysphagia, such as food sticking, cough with eating, globus sensation, or diet change [50]; (2) underlying disease associated with dysphagia, such as stroke, brain tumor, spinal cord injury, and other neurological or neuromuscular illness; (3) dysphagia diagnosed via VFSS; (4) stable vital signs; and (5) written informed consent. Because NMES is reportedly beneficial for swallowing in heterogeneous patient etiologies [50], the inclusion criteria were dysphagic patients with variable etiologies. Exclusion criteria included severe cognitive dysfunction, serious psychiatric disorders, previous cervical surgery, and patients unable to undergo either VFSS or HRM. The clinical characteristics of the participants are presented in Table 3.

**Table 3:** Demographic characteristics

	Total (*n* = 18)	Patient (*n* = 9)	Healthy (*n* = 9)
Age, median (IQR), y		65 (12)	53 (18)
Sex, M:F		8:1	5:4
Diagnosis, No			
Stroke		4	
Brain tumor		1	
Cerebral palsy		1	
Spinal cord injury		1	
Unknown		2	

### Equipment: sequential 4-channel NMES

**Fig. 2:**
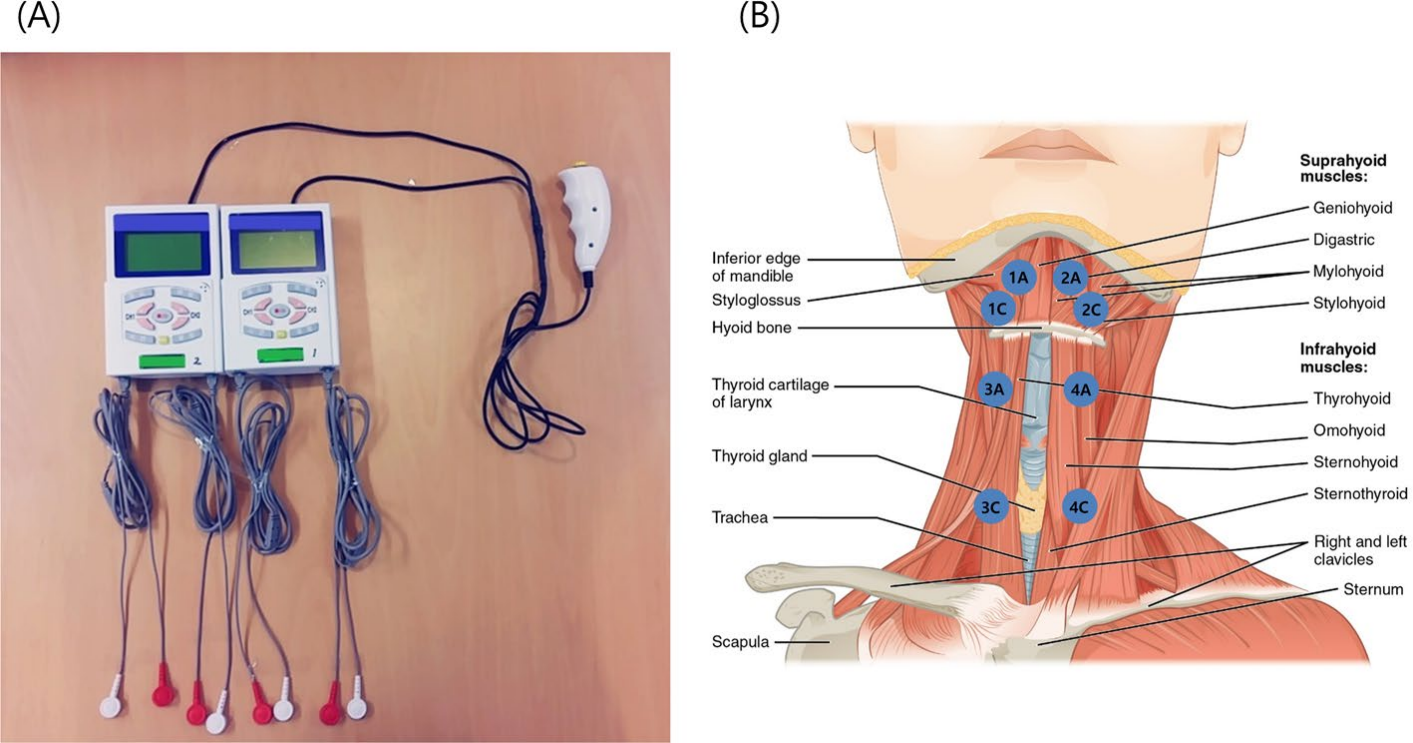
**A** Sequential 4-channel NEMS device was composed of two sets of 2-channel electrical stimulators and uses eight electrodes for electrical stimulation. **B** Locations of the electrode attachments. (Source: https://cnx.org/conte nts/FPtK1 zmh@8.25:fEI3C 8Ot@10/Preface. JW Lim recreated the drawing by adding electrodes to the original)

A sequential 4-channel NMES was newly developed, and its functioning was based on the normal contractile sequence of swallowing-related muscles [14]. This device was composed of two sets of 2-channel NMES devices (Cybemedic Corp., Iksan, Korea) (Fig. 2A). The first NMES device was used for channels 1 and 2, and the second for channels 3 and 4. It also included eight round-shaped electrodes with a diameter of 24 mm (Cybermedic Corp.). Channel 1 (anode electrode 1A and cathode electrode 1C) and channel 2 (anode electrode 2A and cathode electrode 2C) electrodes targeted the bilateral digastric and mylohyoid muscles. Channel 1A and 2A electrodes were placed at the midline of the half point, between the mandible and hyoid bone, with a 1 cm interval; the 1C and 2C electrodes were placed 1 cm lateral to the 1A and 2A electrodes. Channel 3 (anode electrode 3A and cathode electrode 3C) and channel 4 (anode electrode 4A and cathode electrode 4C) electrodes targeted the infrahyoid muscles. Channel 3A and 4A electrodes were placed on the bilateral superior poles of the thyroid cartilage to target the bilateral thyrohyoid muscles, and channel 3C and 4C electrodes were placed medial to the sternocleidomastoid muscle and inferior to the thyroid cartilage. The targeted muscles were the other infrahyoid muscles (sternohyoid, omohyoid, and sternothyroid) (Fig. 2B). The first NMES device was stimulated 300 ms before the second device. The duration of stimulation of the first and second NMES devices were 1400 and 1100 ms, respectively. Hence, all stimulations in the sequence ended concurrently. The pulse frequency was 70 Hz and the biphasic pulse duration was 350 μs. The amplitude of each channel could be independently adjusted (between 0 and 25 mA). The intensity level was further increased until the participants could no longer tolerate discomfort or pain, yielding the maximum tolerance level similar to previous studies [3, 11]. For synchronous stimulation of NMES with swallowing, all participants were required to push the start button and start swallowing simultaneously. To get used to swallowing with NMES, all participants practiced for more than 20 min to become accustomed to swallowing with NMES before starting a recoding.

### Procedure

**Fig. 3:**
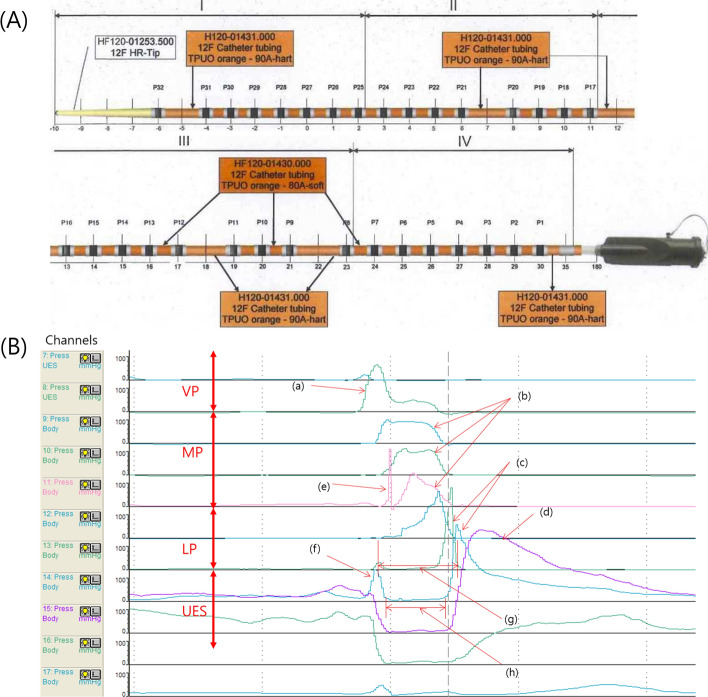
**A** Diagram of the manometric catheter. The total length of the sensor was 36 cm. In most areas of the manometric catheter, sensors spaced at 1-cm interval, and 2 cm in only five areas. **B** Waveform of HRM. The red arrows show the velopharynx (VP), mesopharynx (MP), lower pharynx (LP), and upper esophageal sphincter (UES). **a** velopharyngeal peak, **b** mesopharyngeal peak, **c** inferior pharyngeal constrictor peak, **d** upper esophageal sphincter (cricopharyngeus) peak, **e** tilting of epiglottis, **f** pre-UES peak, **g** UES activity time (interval between the peaks before and after UES relaxation), and **h** nadir UES duration (period of UES relaxation)

A solid HRM (INSIGHT HRIM; Sandhill Scientific, Highlands Ranch, CO) that measured rapidly changing pressure along the entire length of the pharynx in real time was used in this study. In most areas of the manometric catheter, the interval between the sensors was 1 cm, and 2 cm in only five areas; therefore, in most cases, it is possible to measure the timing of contractions of structure at 1 cm intervals. The total length of the sensor was 36 cm and the sensor was unidirectional (Fig. 3A) [24]. The participants were instructed not to consume food or liquids for 4 h and 2 h prior to examination, respectively, to avoid any potential confounding effect of satiety [51]. A 10% lidocaine spray was applied through the nasal passage. The manometric catheter was lubricated with 2% lidocaine jelly to ease its passage through the pharynx. Once the catheter was positioned within the pharynx, the participants rested for 5–10 min for adaptation prior to experimental swallowing [24, 52]. In a neutral head position, all participants underwent HRM evaluation when dry (salivary) swallowing, as follows: 1) before NMES, 2) with NMES, and 3) after NMES. The HRM evaluations were performed two times, and the mean values were used for our study.

### Outcome variables: HRM data analysis

HRM data were extracted using BioVIEW ANALYSIS software (Sandhill Scientific, Version 5.6.3.0). The channels of interest were the velopharynx (VP), mesopharynx (MP), lower pharynx (LP), and upper esophageal sphincter (UES). We measured the pressure, area, and timing variables of these landmarks [24, 25, 51].

VP was defined as the soft palate and posterior pharynx. MP was defined as the tone base and middle pharyngeal constrictors [24, 35, 53]. VP pressure peak (a) and MP pressure peak (b) were easily observed in Fig. 3B. Anatomically, MP was located slightly higher than the epiglottis. A prominent high peak with short duration (Fig. 3Be) was observed occasionally, which was added to the MP. As the epiglottis tilted, it struck the manometric catheter and created a peak with high amplitude and short duration. This peak changed the maximal peak pressure; therefore, the higher-pressure peak of MP should be measured at other channels that are nearby. We selected a MP channel that showed the highest amplitude and highest area between the VP and epiglottis. This channel was usually located 1 channel higher than the peak of the epiglottis. Because the intervals of the channels are 1 cm, these epiglottic peaks were sometimes not visualized. [24, 26]. The mean (SD) values were recorded for maximal pressure and area integral of VP and MP, and onset time of VP pressure peak.

In Fig. 3B, we could observe the narrow peristaltic waves (c) and the last broad peak (d). Anatomically, pharyngeal constrictors are comprised of the superior, middle, and inferior pharyngeal constrictors. In addition, instead of being a true sphincter, the UES is a kidney bean shaped potential space, about 4 cm in length, that is bounded anteriorly by the larynx, posterolaterally by the pharyngoesophageal muscles, superiorly by the pharynx and inferiorly by the esophagus [54]. The cricopharyngeus muscle (CPM), which represents the caudal third of the UES, is the primary intrinsic muscle within the UES, while the IPC are also involved [54]. The IPC is predominantly composed of fast twitch (type II) fibers (39% type I, 61% type II), and the CPM is predominantly composed of slow twitch (type I) fibers (70% type I, 30% type II) [55, 56]. Therefore, in Fig. 3B, the narrow (c) and broad (d) peaks represented the IPC and CPM, respectively. For differentiation, we defined the narrow peak as “IPC peak” and the broad peak as “UES peak.” We measured the maximal peak pressure in the region of the IPC. We also measured the area and maximal pressure of pre-UES peak (Fig. 3Bf); minimal UES pressure; maximal pressure of UES peak (Fig. 3Bd); UES activation time, which was defined as the interval between the pre-UES and post-UES peaks (Fig. 3Bg); UES nadir duration, which was defined as the period of UES relaxation (Fig. 3Bh); and time interval between VP onset and pre-UES peak [24, 47, 49, 57]. In addition, in the calculation of nadir pressure duration, all sensors in the UES were first combined and then the second-order derivative was used to find the onset and offset times of the nadir [45]. We compared these variables between pre- and during NMES (compensatory effects), and between pre- and post- NMES (residual effects).

### Statistical analysis

As a point (x,y) for comparing two probability distributions in Q-Q (quantile–quantile) plots lies on a line, a paired-t test was used to compare outcome variables between pre- and during NMES in each group. Analyses of the outcome variables among the trials in whole participants were undertaken using linear mixed models, with fixed effects of group, time, and group-by-time interaction, and a random effect for patients. Mean differences at during and post-NMES were estimated by the group by time interaction term, with associated 95% CIs and P values. STATA 15.0 software (Stata Corporation, College Station, TX, USA) was used for all statistical analyses. For all analyses, 2-sided *P* < 0.05 was considered statistically significant. The results are presented as the mean (standard deviation).

## Data Availability

The datasets used and/or analyzed during the current study are available from the corresponding author upon reasonable request.

## Abbreviations

HRM: High-resolution manometry
NMES: Neuromuscular electrical stimulation
VP: Velopharynx
MP: Mesopharynx
LP: Lower pharynx
UES: Upper esophageal sphincter
IPC: Inferior pharyngeal constrictor
CPM: Cricopharyngeus muscle
